# Complete Genome Sequence of *Alkalihalobacillus* sp. Strain LMS39, a Haloalkaliphilic Bacterium Isolated from a Hypersaline Lake

**DOI:** 10.1128/mra.00325-22

**Published:** 2022-06-23

**Authors:** Alex Kipnyargis, Romano Mwirichia, Birgit Pfeiffer, Rolf Daniel

**Affiliations:** a Department of Biological Sciences, University of Embu, Embu, Kenya; b Genomic and Applied Microbiology & Göttingen Genomics Laboratory, Institute of Microbiology and Genetics, Georg-August-Universität Göttingen, Göttingen, Germany; University of Delaware

## Abstract

Here, we report the complete genome sequence of a haloalkaliphilic bacterium (*Alkalihalobacillus* sp. strain LMS39) isolated from Lake Magadi, a hypersaline lake in Kenya. The genome comprised 4,850,562 bp with a GC content of 37%.

## ANNOUNCEMENT

Haloalkaliphilic microorganisms are ideal models for studying adaptation to extreme ecosystems (1) and encode biocatalysts that are utilized in industrial applications (2). Strain LMS39 was isolated from dry sediments of Lake Magadi, Kenya (1°43′–2°00′S, 36°13′–36°18′E). Sediment (0.1 g) was serially diluted, and 10^−9^ and 10^−10^ dilutions were spread onto basal agar medium containing peptone (2 g/L), yeast extract (0.5 g/L), K_2_HPO_4_ (1 g/L), CaCl_2_·2H_2_O (0.05 g/L), MgSO_4_·7H_2_O (0.1 g/L), and agar (14 g/L) (3). The medium was prepared with sterile lake water and supplemented with 1% (wt/vol) cellulose. Singularization was conducted by restreaking the strains at least three times. Pure cultures were grown on Trypticase soy broth (TSB) supplemented with NaCO_3_ (1% [wt/vol]) and NaCl (4% [wt/vol]) in a rotary shaker (180 rpm) at 37°C for 12 h.

Chromosomal DNA was extracted using the MasterPure complete DNA and RNA purification kit as recommended by the manufacturer (Epicentre, Madison, WI, USA). Sanger sequencing and analysis of the LMS39 16S rRNA gene sequence revealed 97% identity to that of Alkalihalobacillus bogoriensis sp. strain LBB3 (NR042894). An Illumina paired-end sequencing library was generated using the Nextera XT DNA sample preparation kit. Sequencing was conducted using the MiSeq system and reagent kit v3 (600 cycles) according to the manufacturer’s protocol (Illumina, San Diego, CA, USA). Libraries for Nanopore sequencing were generated using 1.5 μg of isolated DNA without size selection. The 1D genomic DNA sequencing protocol for the MinION device was conducted using the ligation sequencing 1D kit (SQK-LSK109) as recommended by the manufacturer (Oxford Nanopore Technologies, Oxford, UK). After end repair using the NEBNext FFPE repair mix (New England Biolabs, Ipswich, MA, USA), sequencing was performed using a SpotON flow cell Mk I (R9.4) and MinKNOW software v18.12.6 (Oxford Nanopore Technologies). Default parameters were used for all software unless otherwise specified. Trimming and adapter removal was performed using fastp v0.20.1 (4) and Porechop v0.2.4 (https://github.com/rrwick/Porechop) for the Illumina and Nanopore reads, respectively. Potential phiX contamination was removed from the Illumina reads using Bowtie2 v2.3.5.1 (5). After quality assessment, 1,884,551 Nanopore reads (*N*_50_, 5,594 bp) and 2,524,128 Illumina paired-end reads were obtained.

*De novo* genome assembly was conducted using Unicycler v0.5.0 (6) and validated with Bandage v0.8.1 (7), resulting in 1 circular genome (4,850,562 bp) with a GC content of 37.0%. Annotation using PGAP v6.1 (8) yielded 4,749 protein-encoding genes, of which 4,571 had functional assignments. Additionally, genes encoding 89 tRNAs, 30 rRNAs, and 4 noncoding RNAs (ncRNAs) were identified. To assess the relationship between the LMS39 genome and publicly available ones, the type strain genomes available from the Type Strain Genome Server (TYGS) (9) were employed. The average nucleotide identity (ANI) was assessed using the ANIm method in pyani v0.2.11 (10). LMS39 is most closely related to *Alkalihalobacillus bogoriensis* ATCC BAA-922 (GenBank accession number GCA_000621445) with an average nucleotide identity of 92.8%. Accordingly, the strain was designated *Alkalihalobacillus* sp. strain LMS39. Analysis of the carbohydrate-active enzymes (CAZymes) (11) showed that the genome of LMS39 possessed genes encoding cellulase, β-glucosidases, β-xylanase, β-xylosidase α-amylase, neopullulanases, and pectate lyases.

### Data availability.

The annotated genome sequence of *Alkalihalobacillus* sp. strain LMS39 has been submitted to GenBank under the accession number CP093300.1. The raw reads were deposited at the NCBI Sequence Read Archive (SRA) under the accession number SRR18516662 for the Illumina reads and SRR18516661 for the Nanopore reads.
